# Using a Two-Sample Mendelian Randomization Method in Assessing the Causal Relationships Between Human Blood Metabolites and Heart Failure

**DOI:** 10.3389/fcvm.2021.695480

**Published:** 2021-09-14

**Authors:** Zixian Wang, Shiyu Chen, Qian Zhu, Yonglin Wu, Guifeng Xu, Gongjie Guo, Weihua Lai, Jiyan Chen, Shilong Zhong

**Affiliations:** ^1^School of Biology and Biological Engineering, South China University of Technology, Guangzhou, China; ^2^Department of Pharmacy, Guangdong Provincial People's Hospital, Guangdong Academy of Medical Sciences, Guangzhou, China; ^3^Guangdong Provincial Key Laboratory of Coronary Heart Disease Prevention, Guangdong Cardiovascular Institute, Guangdong Provincial People's Hospital, Guangdong Academy of Medical Sciences, Guangzhou, China; ^4^Guangdong Provincial People's Hospital, Guangdong Academy of Medical Sciences, School of Medicine, South China University of Technology, Guangzhou, China; ^5^School of Pharmaceutical Sciences, Southern Medical University, Guangzhou, China

**Keywords:** heart failure, blood metabolites, Mendelian randomization, 4-vinylphenol sulfate, causal relationship

## Abstract

**Background:** Heart failure (HF) is the main cause of morbidity and mortality worldwide, and metabolic dysfunction is an important factor related to HF pathogenesis and development. However, the causal effect of blood metabolites on HF remains unclear.

**Objectives:** Our chief aim is to investigate the causal relationships between human blood metabolites and HF risk.

**Methods:** We used an unbiased two-sample Mendelian randomization (MR) approach to assess the causal relationships between 486 human blood metabolites and HF risk. Exposure information was obtained from Sample 1, which is the largest metabolome-based genome-wide association study (mGWAS) data containing 7,824 Europeans. Outcome information was obtained from Sample 2, which is based on the results of a large-scale GWAS meta-analysis of HF and contains 47,309 cases and 930,014 controls of Europeans. The inverse variance weighted (IVW) model was used as the primary two-sample MR analysis method and followed the sensitivity analyses, including heterogeneity test, horizontal pleiotropy test, and leave-one-out analysis.

**Results:** We observed that 11 known metabolites were potentially related to the risk of HF after using the IVW method (*P* < 0.05). After adding another four MR models and performing sensitivity analyses, we found a 1-SD increase in the xenobiotics 4-vinylphenol sulfate was associated with ~22% higher risk of HF (OR [95%CI], 1.22 [1.07–1.38]).

**Conclusions:** We revealed that the 4-vinylphenol sulfate may nominally increase the risk of HF by 22% after using a two-sample MR approach. Our findings may provide novel insights into the pathogenesis underlying HF and novel strategies for HF prevention.

## Introduction

Heart failure (HF) is a major public health problem and has imposed considerable burden on society (1). Although great progress has been made in current treatment of HF, its morbidity and mortality continue to rise (2). HF is estimated with a heritability of ~26% (3). Previous genome-wide association studies (GWAS) have identified a few genetic loci for HF (4), while its roles in etiology are unclear. As functional intermediates, circulating metabolites can reflect the underlying biological links of the individual genetic composition and the development of diseases. To date, metabolic dysfunction was proposed as an important contributor in HF (5), and metabolomic studies have identified a number of circulating metabolites associated with HF (6, 7). However, the causal relationships between the metabolites and HF are unclear, and translating these metabolic findings into pathophysiological mechanisms and novel therapies is difficult. Hence, a comprehensive analysis is needed to uncover the interactions between genetics and circulating metabolites in the pathogenesis of HF.

The basic idea of Mendelian randomization (MR) is to use genetic variation as an instrumental variable (IV), which is strongly related to exposure factors and can infer the causal effects between exposure factors and research outcomes (8). To date, some MR studies have been performed in exploring the causation between exposure and heart failure, though the main focus was single exposure or routine exposure factors, such as brain natriuretic peptide (9), interleukin-6 (10), and heart rate (11). Few studies focused on the blood metabolites, especially based on the metabolome. A previous study conducted two-sample MR analysis on 486 blood metabolites and five major psychiatric disorders. It has successfully identified several disease-linked metabolites (12), providing novel insights into integrating metabolic mechanism with psychiatric disorders. However, no research about investigating the causal relationships between blood metabolites and the risk of HF has been reported. Hence, we used a two-sample MR approach for assessing the causal relationships between 486 human blood metabolites and risk of HF in this study to provide a deeper understanding of the pathogenesis of HF.

## Methods

### Study Design and Data Resources

The data we used in this study all came from the public dataset, which are publicly available on the database website, and has obtained ethics approval in the previous studies.

**Figure 1:**
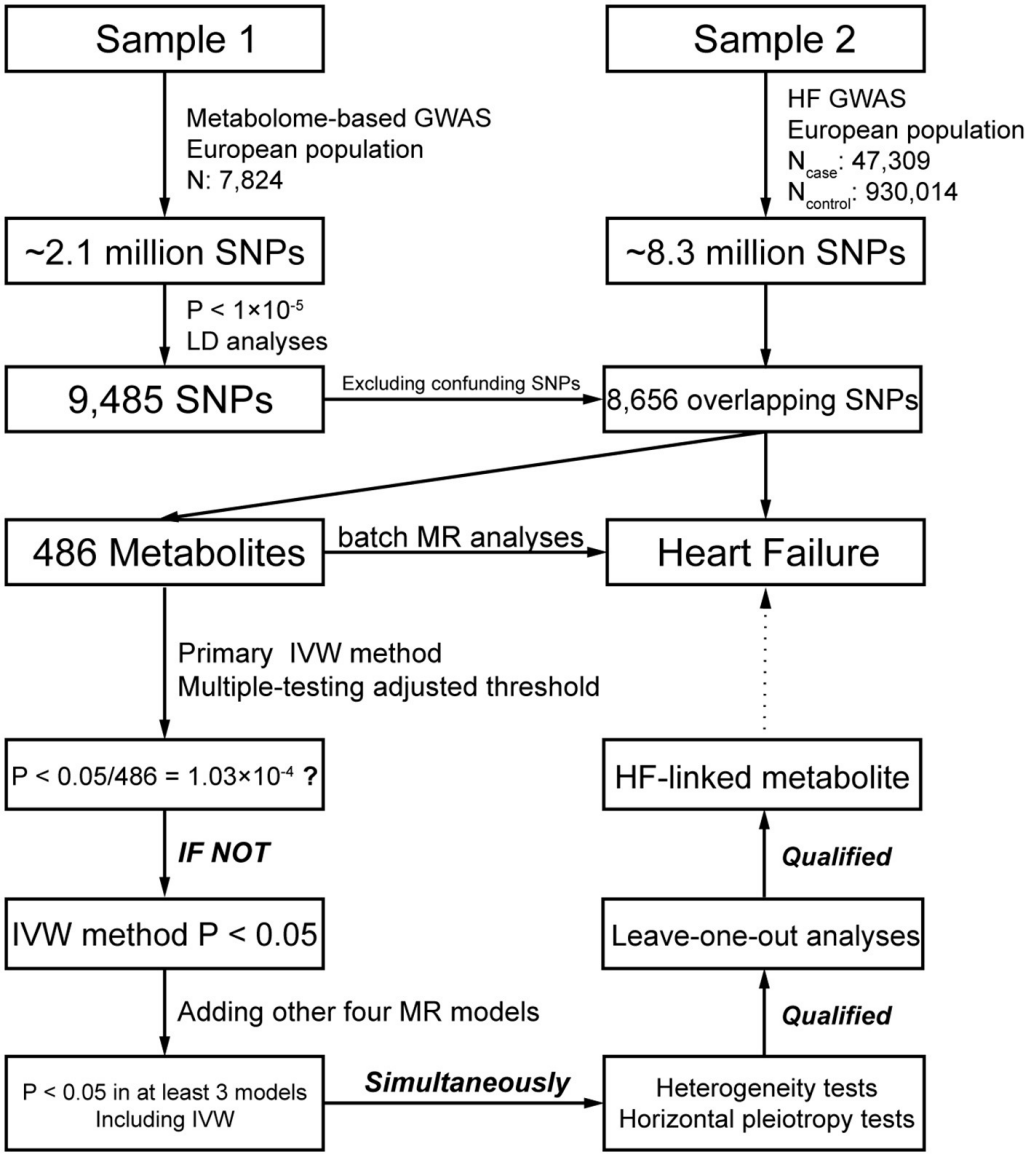
Schematic view of the study design for two-sample MR analyses in this study.

The study flow is illustrated in Figure 1. Exposure information was obtained from Sample 1, which is the largest mGWAS data published by Shin et al. (13) in 2014 and contains 7,824 Europeans. After strict quality control, ~2.1 million single nucleotide polymorphisms (SNPs) and 486 blood metabolites (including 309 known metabolites and 177 unknown metabolites) were employed. These metabolites can be split into eight major categories: carbohydrates, amino acids, nucleotides, cofactors and vitamins, lipids, peptides, energy products, and xenobiotic metabolites. Summary data of all the mGWAS results in Sample 1 are publicly available on a database website (http://metabolomics.helmholtz-muenchen.de/gwas/).

Outcome information was obtained from Sample 2, which is based on the results of a large-scale GWAS meta-analysis conducted by Shah et al. (4) in 2020 on 26 studies of HF. This dataset contains 47,309 cases and 930,014 controls of European lineage, and ~8.3 million SNPs were employed in association analyses. The summary data of HF GWAS in Sample 2 were downloaded from the CVDKP Datasets website (http://www.kp4cd.org/datasets/mi).

### Quality Control of IV

A series of unified selection standards was adopted for the genetic variation in 486 metabolites in this study. We used a relatively loose *P*-value threshold, which was widely used in MR analysis (8), that is, *P* < 1 × 10^−5^, as a significant condition for the preliminary selection of IVs. Then, we performed linkage disequilibrium (LD) analysis to achieve independent genetic instruments, which were derived from a stringent clumping criterion [LD cutoff of *r*^2^ = 0.001 within a 10,000 kb window in the 1000 Human Genomes Project (14) European (EUR) reference panel]. Given that metabolites in similar metabolic pathways may be regulated by the same SNPs and multiple metabolites are significantly associated with the same IVs, so the MR hypothesis could be disturbed. Hence, we conducted the restricted selection of IVs (15) to exclude SNPs that were significantly related to more than two metabolites. Besides, we searched for keywords [(HF) OR (heart failure) AND (SNP) OR (GWAS)] in the PubMed, and we collected SNPs related to HF (including various types, e.g., dilated cardiomyopathy, incident systolic heart failure, advanced heart failure, congestive hearts failure) or its risk factors (such as interleukin-6, ejection fraction, heart rate, aortic root size, etc.) in the published literature. We deleted the disease-related SNPs (Supplementary Table 1) and the duplicate SNPs after sorting and merging. Finally, we used the unique SNP for subsequent analysis.

### MR Analysis

The inverse variance weighted (IVW) model was used as the primary two-sample MR analysis model. IVW was proposed by Burgess et al. (16) and usually used in the MR studies of multiple IVs. This method can be employed on the premises that IVs satisfy the assumptions of relevance, independence, and exclusivity and genetic variation affect outcomes only through exposure in the study. The IVW method is ideal in estimating robust causal detection ability. We considered that the features of these metabolites and HF risk have a strong causal relationship if the *P*-value of IVW exceeds the multiple-testing adjusted threshold (*P* < 0.05/486 = 1.03 × 10^−4^). However, given that the causal effects between blood metabolites and risk of HF sometimes are limited, a strict threshold might lead to the loss of some potential signals. Hence, we focused on potentially causal metabolites (*P* > 1.03 × 10^−4^ but *P* < 0.05) and added four extra MR models to test the causal influence features, namely, MR-Egger regression (17), the weighted median method (18), the simple mode-based estimator (19), and the weighted mode-based estimator (19).

MR-Egger regression is the weighted linear regression of the effects of IVs and exposure and the effects of IVs and outcome (17). Different from the intercept term of IVW forced linear regression, the latter is zero, the intercept term in this model is a variable, and the horizontal pleiotropy of genetic variation can be measured by the intercept term. The fact that the intercept term does not correspond to zero indicates pleiotropy, but MR-Egger can still get an unbiased estimation when the IVs exist pleiotropy, which is its advantage. When applying the MR-Egger model, the tool variables will have nothing to do with the outcome, and only through exposure factors affect the outcome, which weakens the exclusive hypothesis of the IVM method to the tool variables. MR-Egger just needs to meet the hypothesis named “InSIDE (instrument strength independent of direct effect) assumption” that the precise effects of tool variables and outcomes are independent of the correlation between tool variables and exposure factors. It is to be noted that the direction of all tool variables is the same in the analysis. Although the assumption of IVs can be effectively evaluated through the intercept item of MR Egger, it is less effective than IVW approach in detecting the causality (20).

The weighted median method is generally employed in measuring an effect, and the ratios of selected SNPs are calculated for the estimation of a weighted empirical distribution function (18). This method allows a strong SNP to provide an asymptotically consistent estimate of causal effects; even when an effective SNP is less, it can also reduce the bias of causal effects estimation.

The simple mode-based estimator classifies SNPs according to causal effects, and similar values are divided into a cluster. The estimated causal effect is estimated by a cluster with the largest number of SNPs. The weighted mode-based estimator weighs the causal effects values of each SNP pair to the number of SNPs in each cluster, and the results returned are temporary estimates with the maximum number of SNPs weight. The premise of using Mode-based Estimate method to access the consistent estimation of causal effect is to satisfy the “ZEMPA hypothesis” (Zero Modal Pleiotropy Assumption), that is, in the total genetic variation, the mode of the bias term is 0 (19).

In brief, if the five MR models mentioned above produce similar estimates of causal effects and show significant *P*-values (*P* < 0.05) in at least three models (including IVW), then we consider the metabolite as a candidate causal feature for HF risk.

### Sensitivity Analysis

Owing to the diversity of experimental conditions, analytical platforms, and study subjects, there may be heterogeneity in the two-sample MR analyses, resulting in bias in the estimation of causal effects. Thus, heterogeneity testing of IVW analysis and MR-Egger regression was adopted in this study. If the *P* > 0.05 in the test, evidence of heterogeneity in the included IVs is non-existent, that is, the influence of heterogeneity on the estimation of causal effects can be ignored.

When we use IVW to explore the causal relationship, there may be other unknown confounding factors against genetic multiplicity and bias estimation of causal effects. Hence, we performed horizontal pleiotropy test by judging the intercept of MR-Egger regression and evaluating the *P*-value of it on the MR-Egger model. If the intercept is close to 0 (<0.1) and *P* > 0.05, we considered that there is no evidence for the existence of horizontal pleiotropy in the tests. In addition, we adopted MR-PRESSO method to further test horizonal pleiotropy and possible outliers by using MR-PRESSO package (21).

After implementing the heterogeneity test and horizontal pleiotropy test, we used the leave-one-out method in conducting sensitivity analysis on qualified metabolites. In this method, related SNPs are removed one by one, and the amalgamation effect of the remaining SNPs is calculated for the evaluation of the effect of each SNP on the metabolites. If the overall error line does not change considerably after the exclusion of each SNP (i.e., all error lines do not pass through 0), the result is considered reliable.

### Pathway and Enrichment Analysis

We performed pathway and enrichment analysis of 11 HF-related known metabolites (*P* < 0.05, IVW method) through the online metabolomics data analysis website [(22); https://www.metaboanalyst.ca/MetaboAnalyst/faces/home.xhtml]. First, we found the ID corresponding to these metabolites on the Human Metabolome Database [(23); https://hmdb.ca/]. Then, we used Enrichment Analysis and Pathway Analysis modules in the Annotated Features mode to perform the pathway and enrichment analysis. Overall, we collected a number of metabolite sets and pathways of metabolites related to HF based on SMPDB [(24); https://smpdb.ca/] and KEGG database [(25); https://www.kegg.jp/].

### Statistical Analysis

Given that hundreds of exposures were used in this study, batch MR analyses were implemented using our own script. Here, we upload the core codes of batch two-sample MR analysis in GitHub (https://github.com/zxwang2019/Two-sample-MR.git). LD analyses were performed by using the PLINK software (version 1.9) (26). Two-sample MR analyses, including sensitivity analyses, were all performed by using the TwoSampleMR package (version 0.4.22) (27) in R (version 3.6.1).

## Results

### IV Information

A total of 39,142 SNPs were significantly associated with the 486 metabolites (*P* < 1 × 10^−5^) in Sample 1. After LD analyses, the number of these SNPs collapsed into 9,485, and the SNPs were relatively independent from each other. Among the 9,485 SNPs, 335 were associated with at least two metabolites, and no SNP was associated with HF or its risk factors (see Methods). We excluded confounding SNPs and compared them with the SNPs in Sample 2 (Figure 1). Finally, 8,656 (94.6%) SNPs were selected for subsequent analyses. Five metabolites with IV number of less than three or more than 100 were removed in the subsequent MR analyses for stable and reliable statistical results.

### MR Analysis Results

In this study, IVW model was used as the primary method in estimating the causal relationships between the blood metabolites and HF risk. Theoretically, the multiple-testing adjusted threshold (*P* < 1.03 × 10^−4^) was used in assessing significance, and no metabolite exceeded the strict threshold in this study (Supplementary Table 2). A total of 22 metabolites comprising 11 known metabolites and 11 unknown metabolites showed nominally significant relation (*P* > 1.03 × 10^−4^ but *P* < 0.05, IVW method) to HF (Table 1). In the results of the pathway analysis of the 11 known metabolites, we found that the “Valine, leucine, and isoleucine biosynthesis” metabolic pathway that involves L-Isoleucine was significant (*p* = 0.026). L-isoleucine is an essential amino acid and must be supplemented in the diet. A study (28) had shown that the concentration of essential amino acids (including L-isoleucine) in the serum of chronic heart failure patients was significantly lower than that of the control group, suggesting that L-isoleucine may be associated with HF progression. As for the enrichment analysis, however, we did not identify significant (*p* < 0.05) metabolite sets (Supplementary Tables 3–5 and Supplementary Figures 1–3).

**Table 1:** Significant metabolites related to the risk of HF according to IVW results (*P* < 0.05).

**ID**	**Metabolite**	**nSNP**	**Beta**	**SE**	***P***	**OR (95%CI)**
M36098	4-vinylphenol sulfate	10	0.20	0.06	2.16E-03	1.22 (1.07–1.38)
M35187	X-13429	4	0.27	0.09	3.10E-03	1.32 (1.10–1.58)
M34035	Linolenate [alpha or gamma; (18:3n3 or 6)]	3	0.54	0.20	6.31E-03	1.71 (1.16–2.52)
M34336	X-12726	22	0.12	0.05	7.15E-03	1.13 (1.03–1.23)
M35160	Oleoylcarnitine	7	0.47	0.18	8.06E-03	1.60 (1.13–2.28)
M34112	X-12544	18	−0.15	0.06	1.06E-02	0.86 (0.77–0.97)
M01125	Isoleucine	16	0.73	0.29	1.11E-02	2.08 (1.18–3.67)
M16818	X-04495	11	0.35	0.14	1.32E-02	1.42 (1.08–1.87)
M35159	Cysteine-glutathione disulfide	9	−0.19	0.08	1.45E-02	0.82 (0.71–0.96)
M18477	Glycodeoxycholate	7	−0.11	0.05	1.53E-02	0.90 (0.82–0.98)
M33138	X-11793	11	0.30	0.13	1.67E-02	1.35 (1.06–1.73)
M33782	X-10346	14	0.08	0.04	1.81E-02	1.09 (1.01–1.17)
M12768	X-03088	16	−0.30	0.13	2.43E-02	0.74 (0.57–0.96)
M33203	X-11858	15	0.06	0.03	2.92E-02	1.06 (1.01–1.12)
M35186	1-arachidonoylglycerophosphoethanolamine	17	−0.32	0.15	3.05E-02	0.72 (0.54–0.97)
M33192	X-11847	11	0.10	0.05	3.31E-02	1.11 (1.01–1.22)
M11438	Phosphate	4	−0.84	0.40	3.40E-02	0.43 (0.20–0.94)
M35464	X-13671	14	−0.38	0.18	3.45E-02	0.68 (0.48–0.97)
M34530	X-12847	10	0.13	0.06	4.22E-02	1.14 (1.00–1.30)
M33973	Epiandrosterone sulfate	3	0.20	0.10	4.31E-02	1.22 (1.01–1.49)
M01114	Deoxycholate	17	0.11	0.05	4.41E-02	1.11 (1.00–1.24)
M33968	5-Dodecenoate (12:1n7)	11	−0.18	0.09	4.95E-02	0.83 (0.70–1.00)

*nSNP, number of the SNP used for tests; SE, standard error; OR, odds ratio; 95% CI, 95% confidence interval; X, unknown metabolite*.

**Figure 2:**
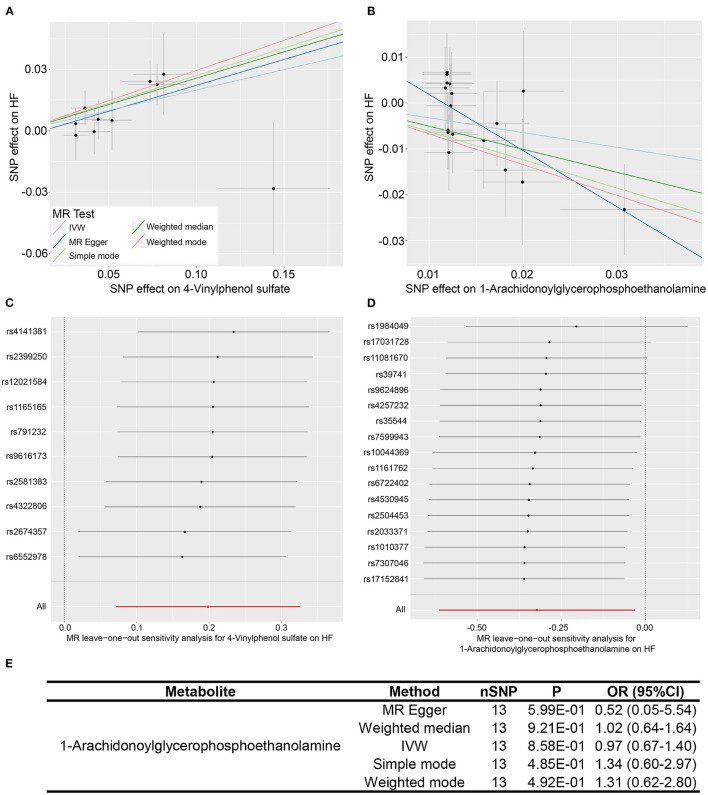
Two metabolites showing causal relationships with HF and their subsequent sensitivity analyses. **(A,B)** Scatter plots of the 5 MR models (light blue line, inverse variance weighted; blue line, MR Egger; light green line, simple model-based estimator; green line, weighted median estimator; red line, weighted model-based estimator) for 4-vinylphenol sulfate and 1-arachidonoylglycerophosphoethanolamine with the risk of HF. **(C,D)** Forest plots show the results of leave-one-out analyses of the two metabolites. **(E)** Re-analyses results of five MR models after the removal of sensitive SNP for 1-arachidonoylglycerophosphoethanolamine.

What we found that 4-vinylphenol sulfate (OR [95%CI], 1.22 [1.07–1.38]), linolenate (alpha or gamma; [18:3n3 or 6]) (OR [95%CI], 1.71 [1.16–2.52]), oleoylcarnitine (OR [95%CI], 1.60 [1.13–2.28]), isoleucine (OR [95%CI], 2.08 [1.18–3.67]), epiandrosterone sulfate (OR [95%CI], 1.22 [1.01–1.49]), and deoxycholate (OR [95%CI], 1.11 [1.00–1.24]) presented potentially increased HF risk, and cysteine-glutathione disulfide (OR [95%CI], 0.82 [0.71–0.96]), glycodeoxycholate (OR [95%CI], 0.90 [0.82–0.98]), 1-arachidonoylglycerophosphoethanolamine (OR [95%CI], 0.72 [0.54–0.97]), phosphate (OR [95%CI], 0.43 [0.20–0.94]), and 5-dodecenoate (12:1n7) (OR [95%CI], 0.83 [0.70–1.00]) presented potentially decreased HF risk. Furthermore, we added four other models (see Methods) to estimate the causal effects between the 11 potentially HF-related metabolites and HF risk (Table 2). Two metabolites were significant in at least three MR models and showed consistent causal effects in all models (Table 2 and Figure 2), namely, 1-arachidonoylglycerophosphoethanolamine (*P*_IVW_ = 3.05 × 10^−2^, *P*_MR Egger_ = 2.48 × 10^−2^, *P*_Weighted median_ = 1.4 × 10^−2^, *P*_Simple mode_ = 1.2 × 10^−1^, *P*_Weighted mode_ = 6.72 × 10^−2^) and 4-vinylphenol sulfate (*P*_IVW_ = 2.16 × 10^−3^, *P*_MR Egger_ = 1.69 × 10^−1^, *P*_Weighted median_ = 3.34 × 10^−3^, *P*_Simple mode_ = 8.7 × 10^−2^, *P*_Weighted mode_ = 2.37 × 10^−2^). For the 4-vinylphenol sulfate, the overall results were similar for the five methods/models. The point estimate from MR-Egger regression was similar to this from IVW, and the interval estimates were relatively wide (Figure 2A). We noted that there may be an outlier here, while the funnel plot (Supplementary Figure 4) showed that the number of points was almost symmetrically distributed when using individual SNPs as IVs (6 vs. 4). But the corresponding causal effect values were less evenly distributed in the IVW and MR-Egger regression models, suggesting that the results obtained using these 10 SNPs as IVs may still be subject to potential bias.

**Table 2:** Five MR models estimate the causal relationships between 11 known metabolites and the risk of HF and tests for heterogeneity and horizontal pleiotropy.

**Metabolite**	**Method**	**nSNP**	***P***	**OR (95%CI)**	***P*_**Heterogeneity**_**	***P*_**Horizontal pleiotropy**_**
4-vinylphenol sulfate	MR Egger	10	1.69E-01	1.29 (0.93–1.79)	0.61	0.74
	Weighted median	10	3.34E-03	1.29 (1.09–1.53)		
	IVW	10	2.16E-03	1.22 (1.07–1.38)	0.7	
	Simple mode	10	8.70E-02	1.31 (0.99–1.72)		
	Weighted mode	10	2.37E-02	1.34 (1.09–1.66)		
Linolenate [alpha or gamma; (18:3n3 or 6)]	MR Egger	3	2.93E-01	2.45 (1.03–5.86)	0.27	0.53
	Weighted median	3	8.30E-03	1.88 (1.18–3.01)		
	IVW	3	6.31E-03	1.71 (1.16–2.52)	0.33	
	Simple mode	3	1.36E-01	1.91 (1.13–3.22)		
	Weighted mode	3	1.22E-01	1.90 (1.17–3.07)		
Oleoylcarnitine	MR Egger	7	6.03E-01	1.81 (0.22–14.86)	0.78	0.91
	Weighted median	7	6.02E-02	1.54 (0.98–2.40)		
	IVW	7	8.06E-03	1.60 (1.13–2.28)	0.87	
	Simple mode	7	4.14E-01	1.36 (0.68–2.73)		
	Weighted mode	7	3.66E-01	1.40 (0.71–2.75)		
Isoleucine	MR Egger	16	2.02E-01	3.11 (0.59–16.39)	0.51	0.62
	Weighted median	16	2.29E-01	1.67 (0.72–3.84)		
	IVW	16	1.11E-02	2.08 (1.18–3.67)	0.57	
	Simple mode	16	7.23E-01	1.33 (0.28–6.20)		
	Weighted mode	16	7.92E-01	1.20 (0.31–4.65)		
Cysteine-glutathione disulfide	MR Egger	9	2.16E-01	0.60 (0.29–1.25)	0.89	0.41
	Weighted median	9	5.26E-02	0.82 (0.67–1.00)		
	IVW	9	1.45E-02	0.82 (0.71–0.96)	0.89	
	Simple mode	9	3.67E-01	0.86 (0.62–1.18)		
	Weighted mode	9	1.98E-01	0.80 (0.59–1.09)		
Glycodeoxycholate	MR Egger	7	8.79E-01	1.03 (0.72–1.46)	0.29	0.46
	Weighted median	7	5.38E-02	0.89 (0.79–1.00)		
	IVW	7	1.53E-02	0.90 (0.82–0.98)	0.33	
	Simple mode	7	1.38E-01	0.87 (0.73–1.02)		
	Weighted mode	7	2.22E-01	0.89 (0.74–1.05)		
1-arachidonoylglycerophosphoethanolamine	MR Egger	17	2.48E-02	0.29 (0.11–0.77)	0.96	0.07
	Weighted median	17	1.40E-02	0.60 (0.40–0.90)		
	IVW	17	3.05E-02	0.72 (0.54–0.97)	0.83	
	Simple mode	17	1.20E-01	0.54 (0.26–1.13)		
	Weighted mode	17	6.72E-02	0.51 (0.26–1.00)		
Phosphate	MR Egger	4	2.14E-01	0.33 (0.10–1.11)	0.17	0.59
	Weighted median	4	7.07E-02	0.44 (0.18–1.07)		
	IVW	4	3.40E-02	0.43 (0.20–0.94)	0.24	
	Simple mode	4	2.31E-01	0.39 (0.11–1.34)		
	Weighted mode	4	1.38E-01	0.43 (0.19–0.98)		
Epiandrosterone sulfate	MR Egger	3	3.94E-01	1.30 (0.90–1.87)	0.07	0.73
	Weighted median	3	1.34E-03	1.27 (1.10–1.47)		
	IVW	3	4.31E-02	1.22 (1.01–1.49)	0.13	
	Simple mode	3	2.36E-01	1.28 (0.96–1.71)		
	Weighted mode	3	8.64E-02	1.27 (1.10–1.47)		
Deoxycholate	MR Egger	17	6.08E-01	1.07 (0.83–1.37)	0.41	0.72
	Weighted median	17	2.18E-01	1.10 (0.94–1.28)		
	IVW	17	4.41E-02	1.11 (1.00–1.24)	0.47	
	Simple mode	17	2.74E-01	1.18 (0.89–1.57)		
	Weighted mode	17	3.11E-01	1.15 (0.89–1.49)		
5-dodecenoate (12:1n7)	MR Egger	11	2.28E-01	0.78 (0.54–1.14)	0.75	0.7
	Weighted median	11	3.68E-02	0.78 (0.61–0.98)		
	IVW	11	4.95E-02	0.83 (0.70–1.00)	0.81	
	Simple mode	11	2.60E-01	0.79 (0.53–1.17)		
	Weighted mode	11	1.48E-01	0.79 (0.60–1.06)		

### Evaluation of the Reliability and Stability of the Results

We performed heterogeneity and horizontal pleiotropy tests on the 11 known metabolites (*P* < 0.05, IVW method) to evaluate the reliability and stability of the results. The *P*-values of the test results (including MR-Egger and MR-PRESSO methods) were more than 0.05 and the intercept of MR-Egger regression is close to 0 (<0.1), suggesting evidence of the existence of heterogeneity and horizontal pleiotropy in these metabolites is non-existent (Table 2 and Supplementary Table 6). As for the two relatively robust metabolites (significant in at least three MR models, 1-arachidonoylglycerophosphoethanolamine, and 4-vinylphenol sulfate), we performed sensitivity analyses by using a leave-one-out approach to test the stability. All IVs (SNPs) of 4-vinylphenol sulfate showed no sensitivity to the results, suggesting a strong link between exposure and outcome, whereas the four IVs (rs1984049, rs17031728, rs11081670, and rs39741) of 1-arachidonoylglycerophosphoethanolamine may have significantly affected the result (Figures 2C,D). After removing the four sensitive SNPs, we performed MR analyses again using the five models, and we found that the results were no longer significant (Figure 2E).

## Discussion

**Figure 3:**
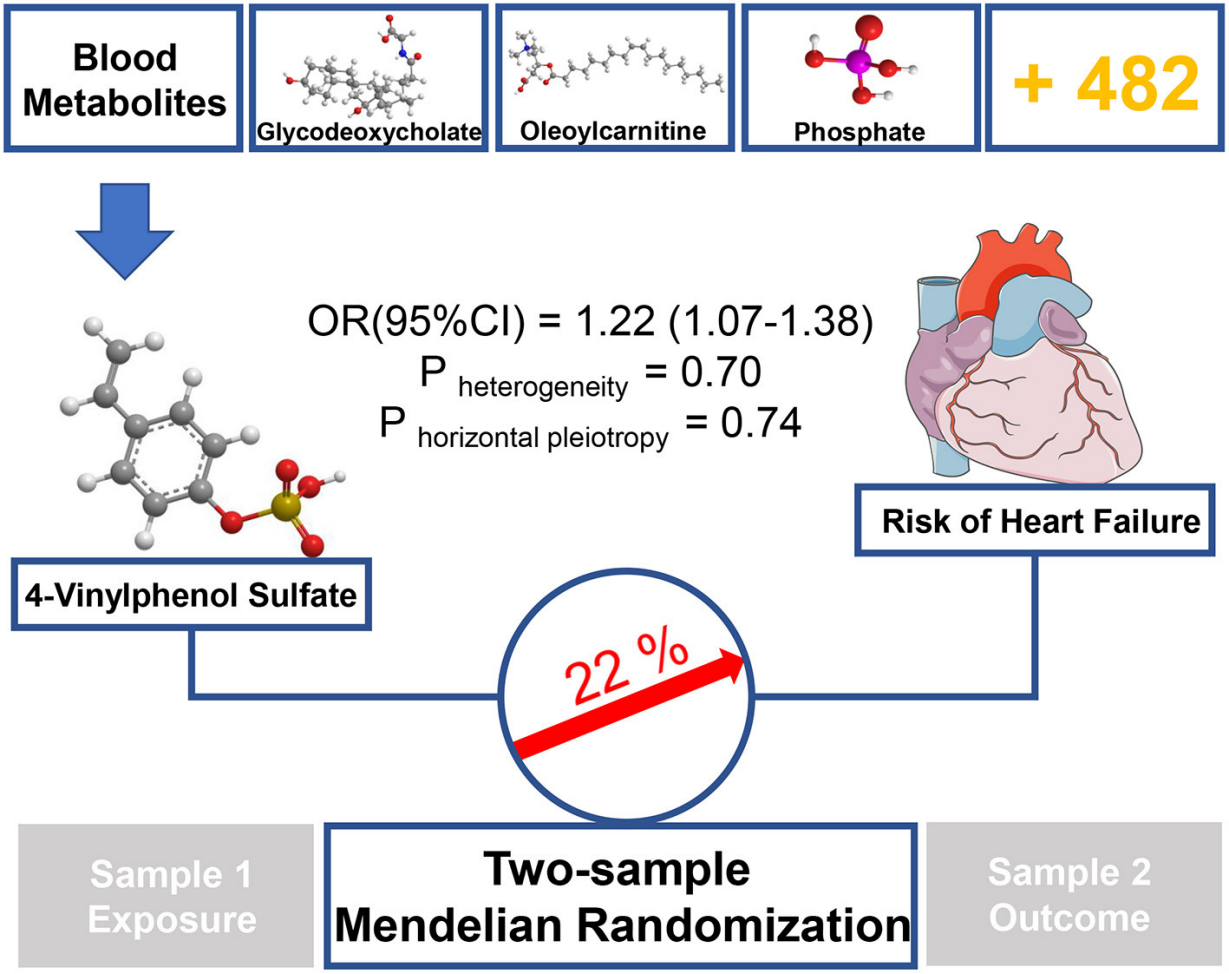
Graphical summary. Among 486 human blood metabolites, this study found that a 1-SD increase in the xenobiotics 4-vinylphenol sulfate was associated with ~22% higher risk of HF by using two-sample MR approach.

In this study, we performed unbiased two-sample MR analysis to perform causal evaluation on 486 blood metabolites and HF risk. We collected the largest mGWAS and large HF GWAS summary data from public databases. We used genetic variants as IVs and discovered 11 known metabolites, which were considered potential risk predictors of HF after primary IVW analysis. Moreover, to further ensure the reliability and stability of the results, another four MR models and sensitivity analysis were performed. The result consistently supported that the xenobiotic 4-vinylphenol sulfate is related to increased HF risk (see Figure 3).

As a sulfate conjugate, 4-vinylphenol sulfate is one of the main metabolisms of 4-vinylphenol *in vivo* (29). Naturally found in crops, such as peanut and wild rice (30), 4-vinylphenol is an essential ingredient widely used in meat and seafood flavor formulations (PubChem CID: 62453). Our findings showed that 4-vinylphenol sulfate could increase the incidence of HF by 22% (IVW method), suggesting that long-term or excessive diets containing such compound or 4-vinylphenol, especially in the additives, may increase the likelihood of HF. Previous studies have shown that the level of 4-vinylphenol sulfate in the blood is closely related to smoking (31), which is a key risk factor for myocardial systolic dysfunction and hospitalization due to mental failure (32). Petersen et al. (33) showed a significant correlation between 4-vinylphenol sulfate and methylation at a certain site of *RARA*, which is a transcription factor that regulates differentiation and apoptosis (34), and the evidence may be linked to the pathogenesis of HF.

Another metabolite is worth mentioning, namely, 1-arachidonoylglycerophosphoethanolamine, which can be referred to as LysoPE [20:4 (5Z,8Z,11Z,14Z)/0:0] or LPE (20:4/0:0). After analysis by the primary IVW method, it is found that the metabolite was related to decreased risk of HF and showed a significant (*P* < 0.05) causation with HF in another two MR models. However, it did not pass the final leave-one-out analysis. LysoPE [20:4 (5Z,8Z,11Z,14Z)/0:0] is an endogenous compound and a kind of lysolipid. Gao et al. (35) found that LysoPE 20:4 is significantly related to Qi deficiency syndrome in the treatment of congestive HF with traditional Chinese medicine, suggesting that it may be one of the specific metabolic biomarkers of congestive HF treated using traditional Chinese medicine granules. In addition, HF is associated with significant disturbances in phospholipid metabolism. A statistically significant decline in LysoPE level was found in patients with chronic HF with reduced ejection fraction (36). Supplementation with LysoPE in mammalian cells can reverse mitochondrial impairments (37). Our findings suggested that LysoPE 20:4 has a potential positive influence on HF risk, providing an interesting and valuable evidence for future studies.

### Innovations and Limitations

Our study has some innovations. First, from the perspective of molecular mechanism, regarding blood metabolites as exposure factors in exploring the causal relationships between metabolites and HF risk has a solid theoretical basis and important clinical research value. Second, the study used strict quality control conditions and reasonable analysis methods, including a variety of models, to evaluate the causal effects. Thus, the results of this study are reliable and stable. Third, unlike in previous MR analyses of single exposure factors, analysis of a large number of blood metabolites may require huge workloads and present analytical challenges. The analysis strategy we presented might provide a reference for similar studies. Our study may have some limitations. To begin with, all the mGWAS and HF GWAS data were obtained from the European population, and thus comprehensive studies involving different ethnic groups are needed. Furthermore, half of the risk predictors of HF obtained by preliminary analysis (IVW only) are unknown metabolites, and their functional structures are unclear. Thus, the findings in the study are limited. Finally, although we revealed that 4-vinylphenol sulfate is nominal causal related to heart failure by using an unbiased two-sample MR approach, while this relationship was theoretical and we failed to confirm it mechanistically. Hence, further work is still needed to uncover the role of 4-vinylphenol sulfate in the pathogenesis of HF, therefore confirming this causal relationship.

## Conclusions

In conclusion, we used a two-sample MR approach to explore the causal relationships between 486 blood metabolites and HF among more than 0.9 million Europeans. We found that 1-SD increase in the xenobiotic 4-vinylphenol sulfate could nominally increase the risk of HF by 22%. Our findings strengthen our knowledge of the relationships between blood metabolites and HF, which potentially facilitate the establishment of personalized explanation or markers for biological differences in disease status.

## Data Availability Statement

Publicly available datasets were analyzed in this study. This data can be found here: the Metabolomics GWAS Server: http://metabolomics.helmholtz-muenchen.de/gwas/; the CVDKP Datasets: http://www.kp4cd.org/datasets/mi.

## Author Contributions

ZW designed the study, performed data analysis, and drafted the manuscript. SC performed data analysis, drafted, and revised the manuscript. QZ revised the manuscript. YW, GX, and GG collected the data. WL and JC provided the resources. SZ designed the study, leaded the study, and revised the manuscript.

## Funding

This work was supported by the grants from the National Nature Science Foundation of China (Nos. 81872934 and 81673514), the Key research and development program of Guangdong Province, China (No. 2019B020229003), Science and Technology Development Projects of Guangdong Province, China (No. 2017B0303314041), and Guangdong Provincial People's Hospital Clinical Research Fund (Y012018085).

## Conflict of Interest

The authors declare that the research was conducted in the absence of any commercial or financial relationships that could be construed as a potential conflict of interest.

## Publisher's Note

All claims expressed in this article are solely those of the authors and do not necessarily represent those of their affiliated organizations, or those of the publisher, the editors and the reviewers. Any product that may be evaluated in this article, or claim that may be made by its manufacturer, is not guaranteed or endorsed by the publisher.

## Abbreviations

HF: heart failure
MR: Mendelian randomization
mGWAS: metabolome-based genome-wide association study
GWAS: genome-wide association study
IV: instrumental variable
SNP: single nucleotide polymorphism
LD: linkage disequilibrium
IVW: inverse variance weighted.
