# Supplementary material for: Using a Two-Sample Mendelian Randomization Method in Assessing the Causal Relationships Between Human Blood Metabolites and Heart Failure
Source: Front Cardiovasc Med. 2021 Sep 14;8:695480. doi: 10.3389/fcvm.2021.695480 (PMC8476837; doi:10.3389/fcvm.2021.695480)
Supplement: Supplementary file 1 [file Data_Sheet_1.DOCX]

Supplementary Material

## Supplementary Figures


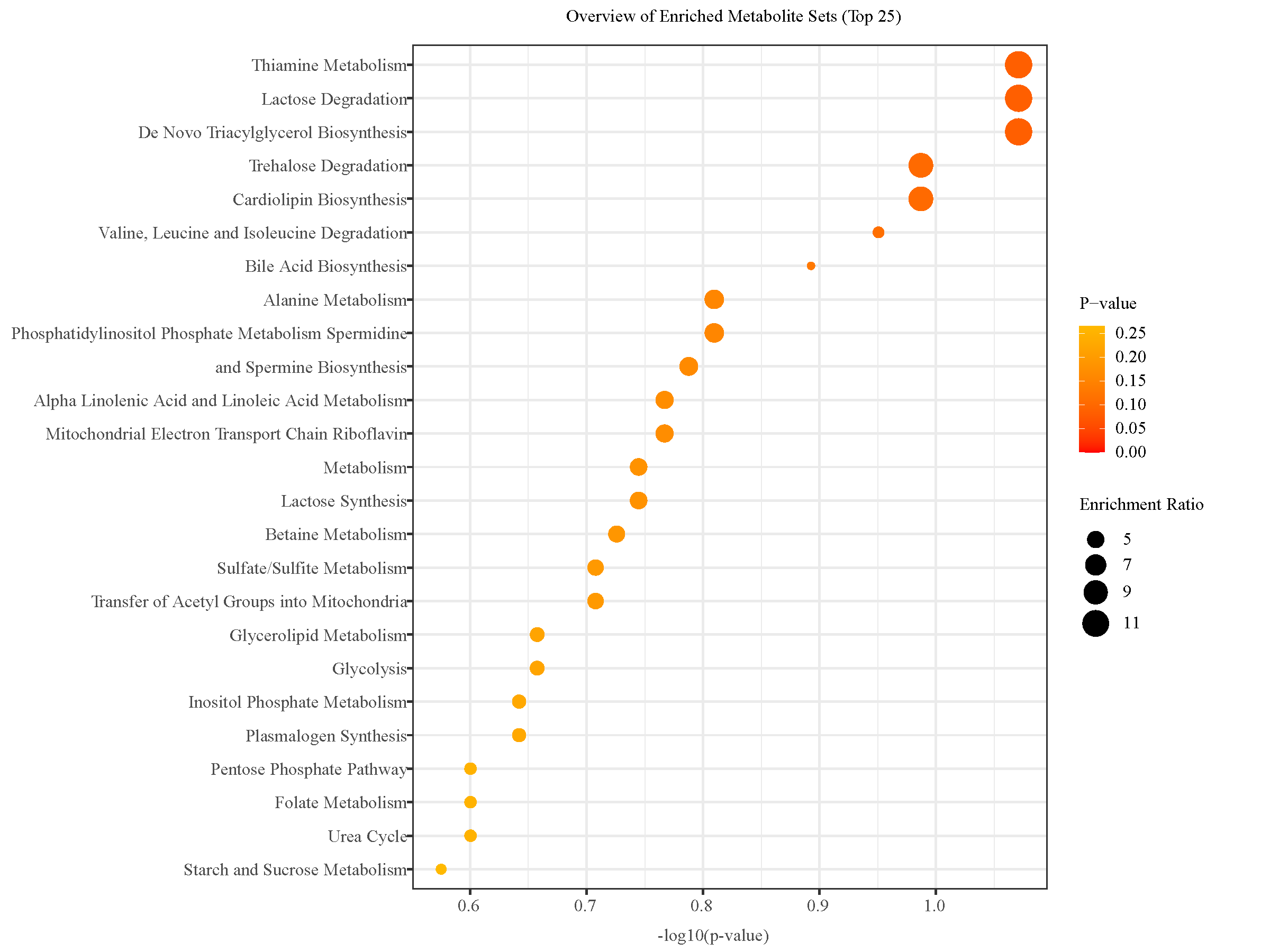


**Figure S1 SMPDB pathway enrichment of** **HF-related metabolites.** This figure shows the top 25 items.


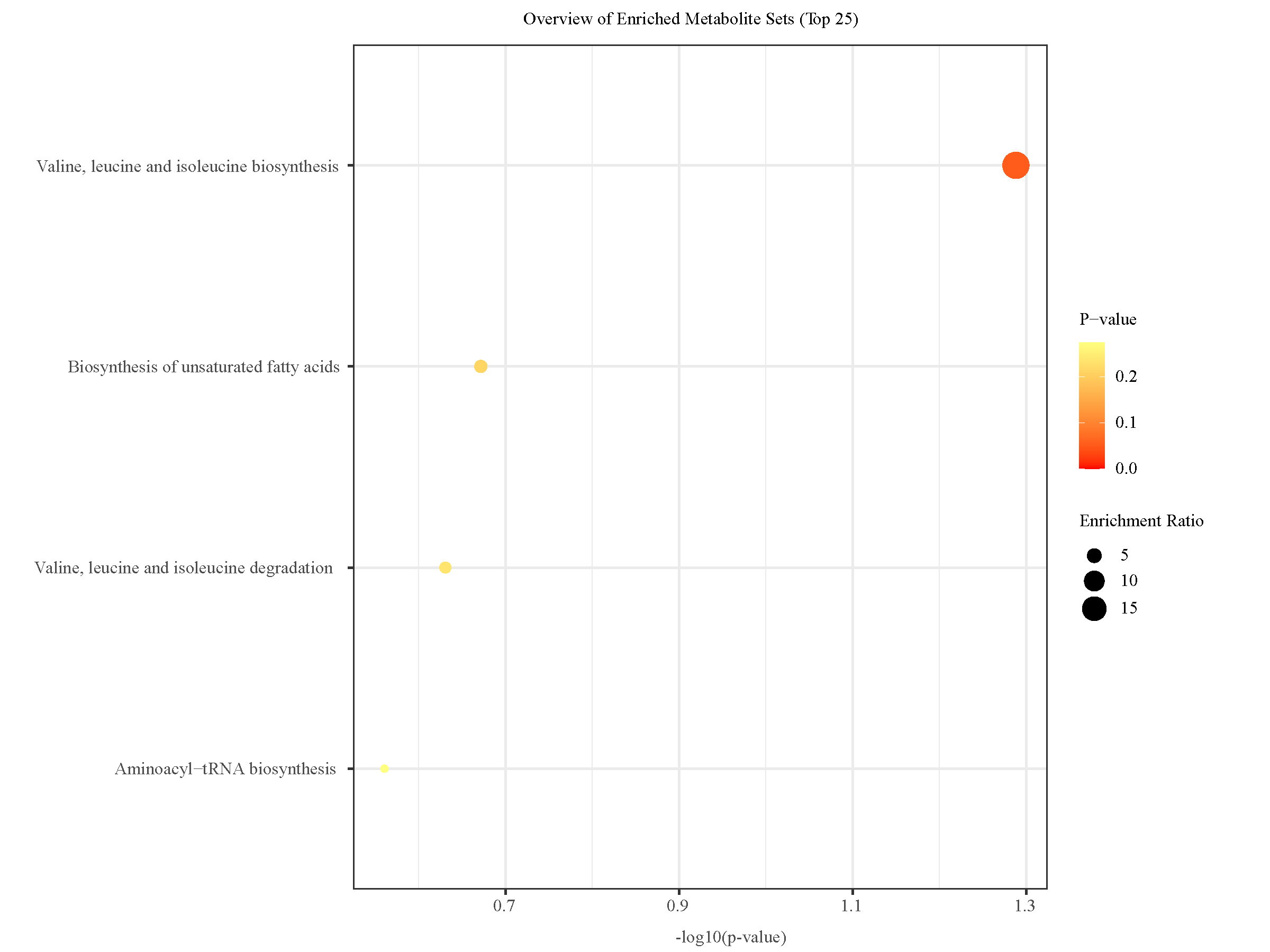


**Figure S2 KEGG pathway enrichment of HF-related metabolites.** This figure shows the only 4 items.


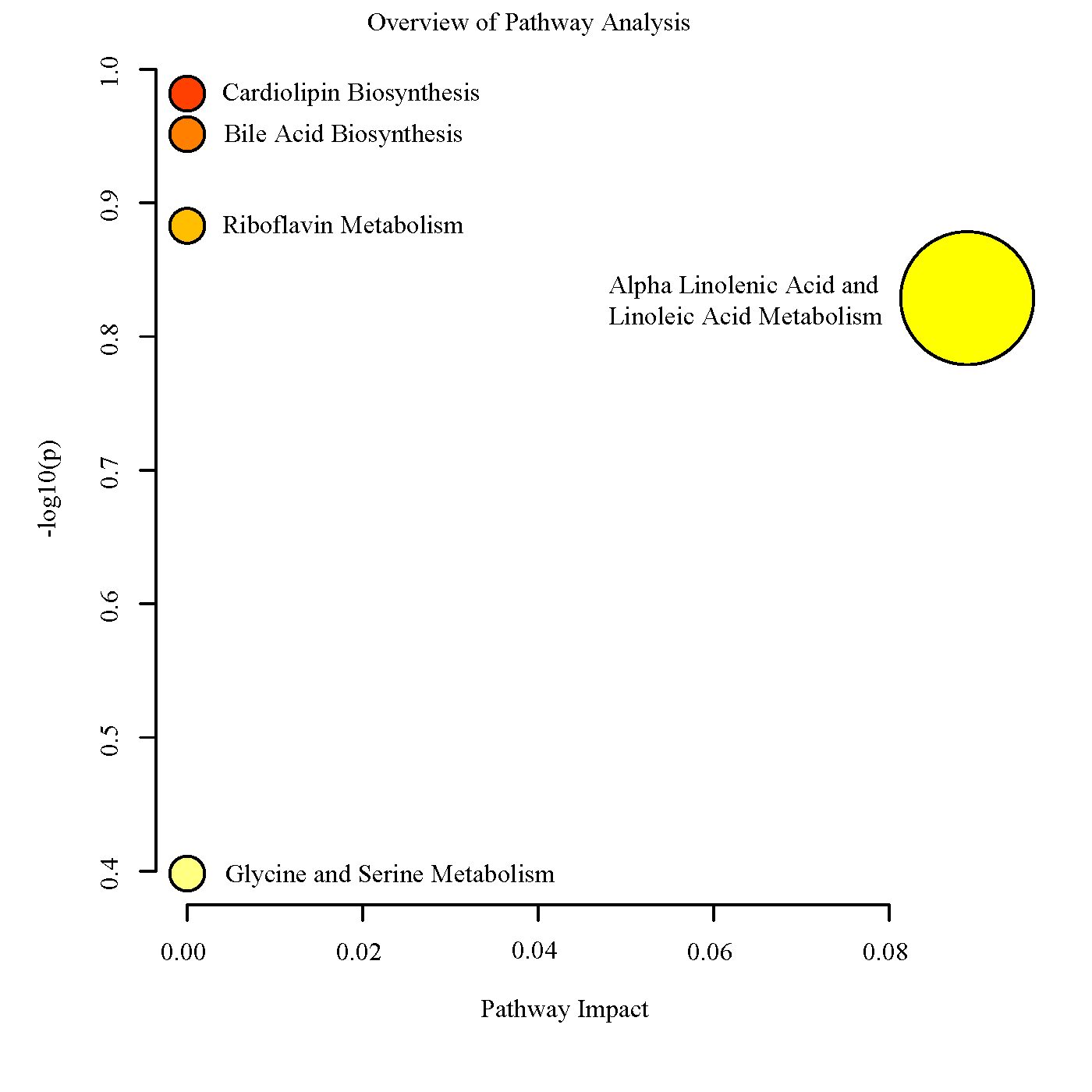


**Figure S3** **Pathway Analysis of HF-related metabolites.** The metabolome view shows all matched pathways according to the p values from the pathway enrichment analysis and pathway impact values from the pathway topology analysis.


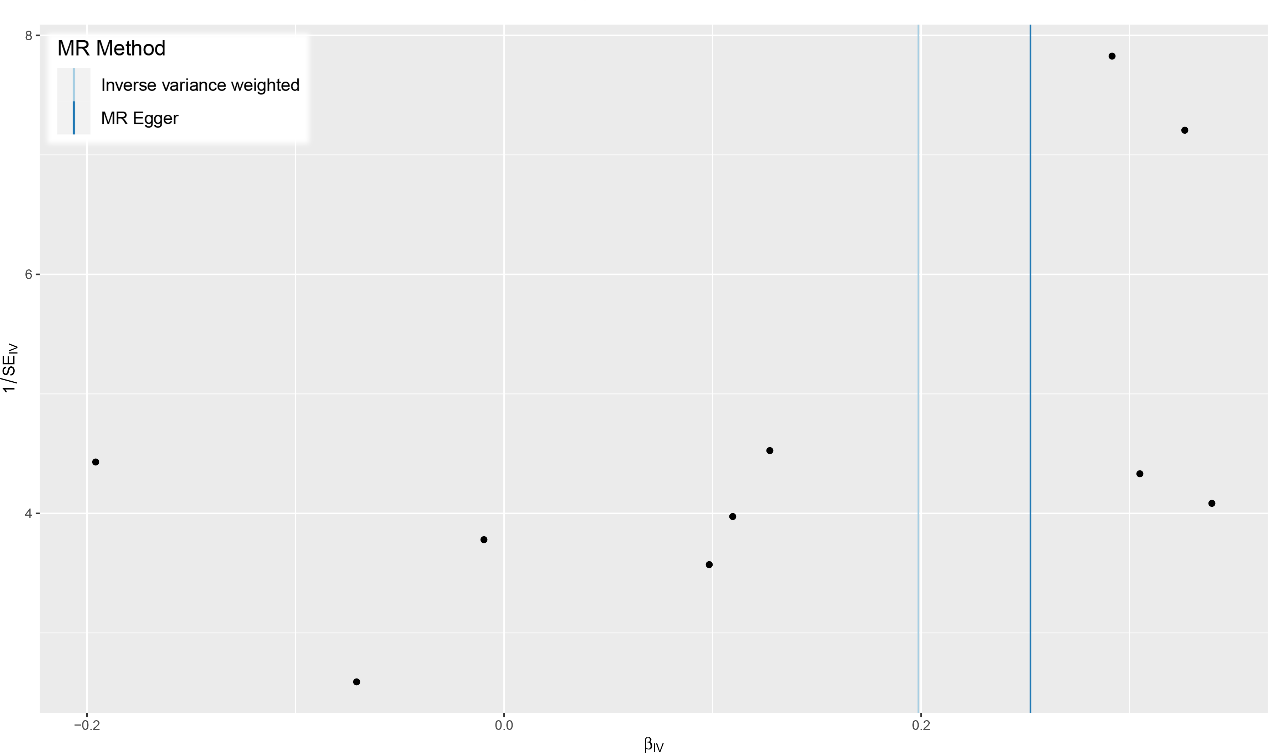


**Figure S4 Funnel plot of Two-sample MR analysis based on the IVW and MR-Egger models with 4-vinylphenol sulfate**
